# Characterization of plant growth-promoting rhizobacteria from perennial ryegrass and genome mining of novel antimicrobial gene clusters

**DOI:** 10.1186/s12864-020-6563-7

**Published:** 2020-02-12

**Authors:** Zhibo Li, Chunxu Song, Yanglei Yi, Oscar P. Kuipers

**Affiliations:** 1 Department of Molecular Genetics, Groningen Biomolecular Sciences and Biotechnology Institute, University of Groningen, Groningen, the Netherlands; 2 College of Resources and Environmental Sciences; National Academy of Agriculture Green Development; Key Laboratory of Plant-Soil Interactions, Ministry of Education, China Agricultural University, Beijing, 100193 China; 3 College of Food Science and Engineering, Northwest A&F University, Yangling, Shaanxi China

**Keywords:** *Bacillus*, *Brevibacillus*, Plant growth-promoting rhizobacteria, Biosynthetic gene clusters, Nonribosomal peptides, Polyketides, Bacteriocins

## Abstract

**Background:**

Plant growth-promoting rhizobacteria (PGPR) are good alternatives for chemical fertilizers and pesticides, which cause severe environmental problems worldwide. Even though many studies focus on PGPR, most of them are limited in plant-microbe interaction studies and neglect the pathogens affecting ruminants that consume plants. In this study, we expand the view to the food chain of grass-ruminant-human. We aimed to find biocontrol strains that can antagonize grass pathogens and mammalian pathogens originated from grass, thus protecting this food chain. Furthermore, we deeply mined into bacterial genomes for novel biosynthetic gene clusters (BGCs) that can contribute to biocontrol.

**Results:**

We screened 90 bacterial strains from the rhizosphere of healthy Dutch perennial ryegrass and characterized seven strains (*B. subtilis* subsp. *subtilis* MG27, *B. velezensis* MG33 and MG43, *B. pumilus* MG52 and MG84, *B. altitudinis* MG75, and *B. laterosporus* MG64) that showed a stimulatory effect on grass growth and pathogen antagonism on both phytopathogens and mammalian pathogens. Genome-mining of the seven strains discovered abundant BGCs, with some known, but also several potential novel ones. Further analysis revealed potential intact and novel BGCs, including two NRPSs, four NRPS-PKS hybrids, and five bacteriocins.

**Conclusion:**

Abundant potential novel BGCs were discovered in functional protective isolates, especially in *B. pumilus*, *B. altitudinis* and *Brevibacillus* strains, indicating their great potential for the production of novel secondary metabolites. Our report serves as a basis to further identify and characterize these compounds and study their antagonistic effects against plant and mammalian pathogens.

## Background

Perennial ryegrass (*Lolium perenne*) is one of the most important pasture plants in the world due to its high levels of palatability and nutritional value for ruminants [1]. The biomass and quality of perennial ryegrass are very crucial for the food chain of grass-ruminant-human since it not only produces food to ruminants but also determines the quality of meat and dairy products for human beings [2, 3]. The susceptibility of plants and potential causes of ruminant diseases by plant-originated pathogens are threats to the safety of this food chain. Although chemical fertilizers and pesticides can ensure the biomass production and the quality of the perennial ryegrass (if not vestigial), their usage may cause severe environmental problems. Thus, there is a need to find an environmentally friendly way to ensure the production of healthy grass.

Plant growth-promoting rhizobacteria (PGPR) have been widely reported to be effective in stimulating the growth of plants as well as protecting the plants from pathogens, which could be an alternative for chemical fertilizers and pesticides. *Bacillus* is one of the most famous PGPR because of its endospore-forming capability, which confers them better survival in the environment [4], and abundant plant growth-promoting traits, including nitrogen fixation, phosphorus solubilization, induced systemic resistance (ISR) induction, and most importantly antimicrobial production [5, 6].

Antimicrobials produced by *Bacillus* and closely related species are very diverse [7]. Based on their biosynthesis pathway, these antimicrobials are classified into three main groups: nonribosomal peptides (NRPs), polyketides (PKs), and bacteriocins. NRPs are synthesized in a nonribosomal pathway through nonribosomal peptide synthetases (NRPSs), which are huge enzymes constituted by different modules. Each module incorporates one amino acid residue, including non-proteinic amino acids. NRPs such as surfactin, fengycin, bacillomycin D, polymyxin, fusaricidin, etc. are very well-known antimicrobials produced by different *Bacillus* and *Paenibacillus* strains [8–10]. PKs are another class of antimicrobials synthesized with mega enzymes, which are called polyketide synthetases (PKSs). Well-known PKs produced by *Bacillus* or *Brecvibacillus* include difficidin, bacillaene, macrolactin, basiliskamides, etc. [11–13]. Contrary to NRPs and PKs, bacteriocins are a class of antimicrobials synthesized in a ribosomal pathway. *Bacillus*-originated bacteriocins such as subtilosin A, plantozolicin, and subtilomycin are well studied [14–16].

Antimicrobials produced by *Bacillus* and closely related species were reported playing very important roles in biocontrol. The abolishment of surfactin production in *Bacillus subtilis* 6051 reduced its colonization to *Arabidopsis* roots and suppression of *Pseudomonas*-originated disease [17]. Iturins and fengycins produced by *B. subtilis* contribute to antagonism against *Podosphaera fusca*, a pathogen cause phyllosphere diseases in melon leaves [18]. *Bacillus velezensis* FZB42 (formerly *Bacillus amyloliquefaciens* FZB42), the Gram-positive model bacterium in biocontrol, employs difficidin, bacilysin, and bacillaene to suppress fire blight disease of orchard trees [19]. In addition, siderophores (bacillibactin), cyclic lipopeptides (surfactin, fengycin, fusaricidin, etc.) can elicit induced systemic resistance (ISR) of plants, thus arming the plant against diseases caused by pathogens [5, 8, 20, 21]. In recent decades, volatiles such as 2,3-butanediol produced by *Bacillus* were also found to be elicitors of ISR [22].

Even though abundant studies focus on *Bacillus* and closely related PGPR, most of them are limited to the plant pathogens and neglect the mammalian pathogens that may enter the body of animals through grazing. For example, *Claviceps purpurea f. secalis*, a fungal pathogen that causes ergotism in ruminants and humans [23], is originated from forage plants. *Pithomyces chartarum*, a fungal pathogen produces sporidesmin that causes facial eczema in sheep [24], also originates from the grass. PGPR that can antagonize both phytopathogens and mammalian pathogens would ensure the safety of this food chain. Therefore, we aimed to isolate and screen *Bacillus* sp. and closely related PGPR strains from the rhizosphere of healthy perennial ryegrass and further mine into the genomes of the candidate PGPR strains to find novel biosynthetic gene clusters (BGCs) that are potentially involved in phytopathogen and plant-originated mammalian pathogen antagonism.

## Results and discussion

### Characterization of strains

**Fig. 1:**
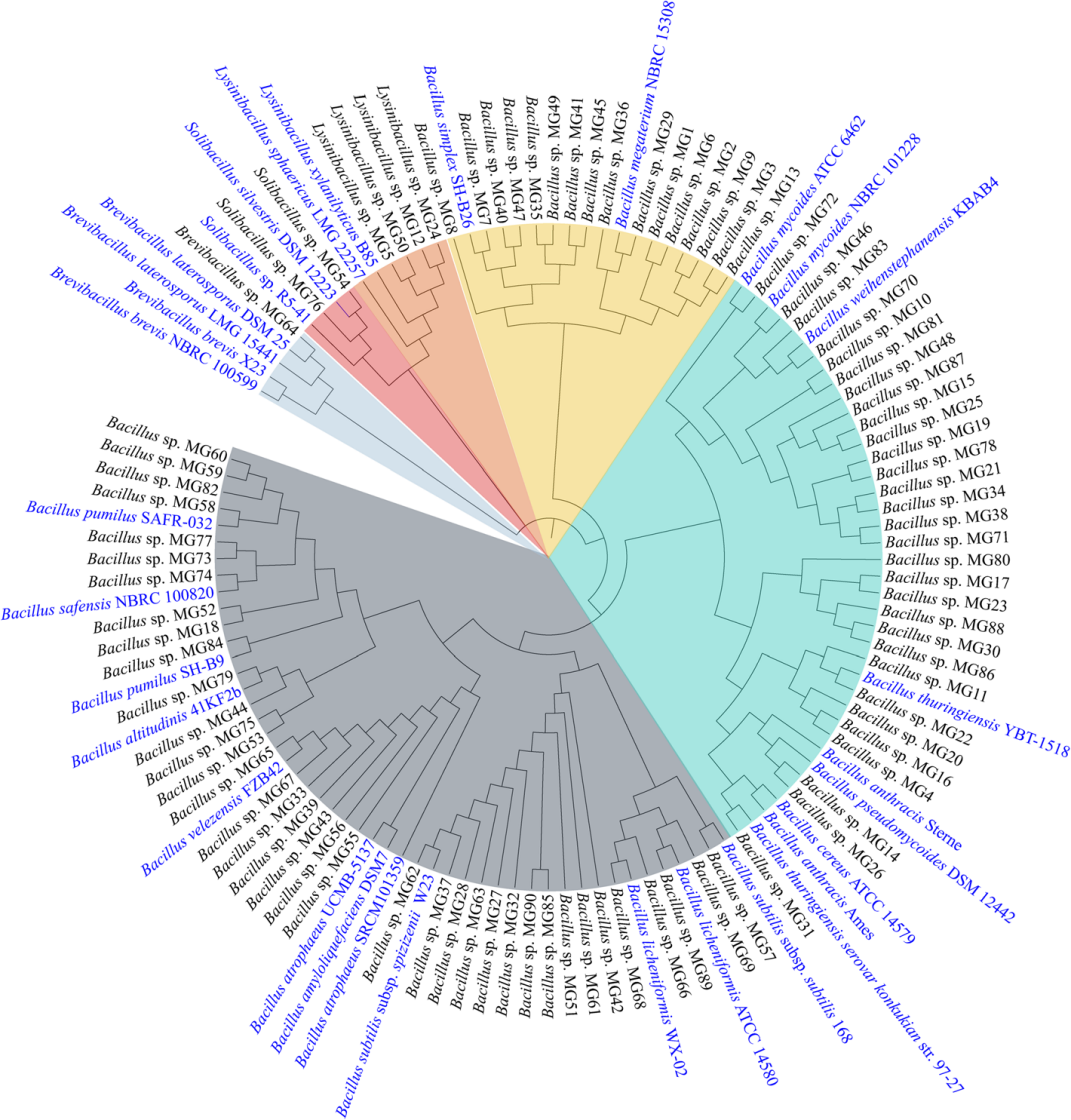
Phylogenetic analysis of the 90 bacterial isolates in this study. Neighbor-joining phylogenetic tree based on partial 16S rRNA sequences was constructed with MEGA7 [26]. The sequences of reference strains were retrieved from the NCBI database. The reference strains are highlighted in blue and different groups were shaded with different colors

A total of 90 Gram-positive bacterial strains were isolated from the rhizosphere of perennial ryegrass [25]. To characterize the strains, 16S rRNA genes were amplified and sequenced. A phylogenetic tree was constructed with the obtained sequences as well as 16S rRNA sequences of representative strains (Fig. 1). The strains were clustered into 4 different genera: *Bacillus* (83 strains), *Lysinibacillus* (4 strains), *Solibacillus* (2 strains), and *Brevibacillus* (1 strain). Among the dominant genus of *Bacillus*, 37 and 30 strains belong to the *B. subtilis* and *B. cereus* groups, respectively, while the rest 16 strains form an independent group that consists of *B. megaterium* and *B. simplex*. The great abundance of *Bacillus* is consistent with the study by Garbeva et al. [27], in which up to 95% of Gram-positive bacteria in the permanent grassland are *Bacillus* and related species.

### Antimicrobial activity

We evaluated all 90 strains for their antimicrobial activity against two pathogens: *Xanthomonas translucens* pv. *graminis* LMG587 and *Magnaporthe oryzae* Guy11. The rationale behind the selection is to cover the two major classes of pathogens (i.e. Gram-negative bacteria and fungi) in perennial ryegrass. *X. translucens* pv. *graminis* is a Gram-negative phytopathogen that causes bacterial wilt in perennial ryegrass and leads to great loss in temperate grassland regions [28]. *M. oryzae* is a fungal pathogen that causes severe blast disease in many *Poaceae* plants, including perennial ryegrass [29, 30]. In total 30 strains showed inhibition against *X. translucens* pv. *graminis* LMG587 and 23 strains against *M. oryzae* Guy11. Of all strains, 16 showed antagonistic activity against both pathogens and 15 of them belong to the *B. subtilis* group, while the last one belongs to the genus of *Brevibacillus* (Additional file 1: Table S1). A total of 7 most inhibitory strains (i.e. MG27, MG33, MG43 MG52, MG64, MG75, and MG84) were selected to extend their antimicrobial spectrum and for further evaluation of their plant growth promotion potential.

Soil-borne plant pathogens and animal pathogens widely exist and cause serious diseases in higher organisms [31, 32]. Some animal pathogens can first adapt to the plant host and finally transmit to animals [33]. Plant pathogens and plant-originated mammalian pathogens that are a potential threat to perennial ryegrass and mammals in the food chain of grass-ruminant-human, were selected as indicators for the antimicrobial activity test. As indicated in Table 1, MG27, MG33, MG43, and MG64 showed very broad inhibitory spectra. They can antagonize Gram-positive bacteria, Gram-negative bacteria, fungi, and oomycetes (Table 1). In contrast to that, MG52, MG75, and MG84 showed better activity on bacteria than on fungi and oomycetes (Table 1). It is worth to note that some of the selected strains displayed antimicrobial activity against the fungal mammalian pathogens. MG27, MG33, MG43, and MG64 can antagonize *C. purpurea f. secalis* and *P. chartarum*, while MG75 and MG84 showed activity against *C. purpurea f. secalis*. This result suggests the possibility of using PGPR to control animal pathogens, thus safeguarding the food chain of grass-ruminant-human. However, we also realize the current knowledge gaps in this area. The mechanisms underlying the interaction of animal pathogens, plants, and PGPR are largely unknown. How to employ PGPR to control animal pathogens in the natural environment is a big question need to be answered. More in-depth studies will be needed before its application.

**Table 1:** Antimicrobial activity of the seven selected bacterial strains from perennial ryegrass

Pathogen types	Species or strains	MG27	MG33	MG43	MG52	MG64	MG75	MG84
Gram-negative bacteria	*Erwinia carotovora* subsp. *brasiliensis* LMG21371	+	+	+	–	–	–	–
	*Pectobacterium carotovorum* LMG05863	+	+	+	–	+	–	–
	*Pseudomonas syringae* pv*. antirrhini* LMG02131	+	+	+	++	+	+	++
	*Pseudomonas syringae* pv. *tomato* DC3000	+	+	+	++	+	+	++
	*Ralstonia syzygii* subsp. *syzygii* LMG06969	+	+	+	–	+	–	–
	*Xanthomonas campestris* pv. *campestris*NCCB92058	+	++	++	++	+	+	++
	*Xanthomonas translucens* pv. *graminis*LMG587^*^	+	++	++	++	++	++	++
Gram-positive bacteria	*Streptomyces scabies* ISP5078	+	+	+	++	+	–	++
Fungi	*Botrytis cinerea*	●	●	●	○	●	○	○
	*Fusarium culmorum*	●	●	●	○	●	○	○
	*Fusarium oxysporum*	●	●	●	○	●	○	○
	*Rhizoctonia solani*	●	●	●	○	●	●	●
	*Magnaporthe oryzae* Guy 11^*^	●	●	●	○	●	○	○
	*Pithomyces chartarum* CBS485.71^#^	●	●	●	○	●	○	○
	*Claviceps purpurea f. secalis* CBS112.45^#^	●	●	●	○	●	●	●
Oomycetes	*Pythium aphanidermatum*	●	●	●	○	●	○	○
	*Pythium ultimum*	●	●	●	○	●	○	○

In the antibacterial assay, no inhibition (−), inhibitory zone < 5 mm (+), inhibitory zone ≥5 mm (++). In the antifungal/oomycetal assay, no inhibition (○), clear inhibition (●).

^*^Pathogens used to screen the isolated strains

^#^Mammalian pathogens

### Plant growth-promoting effect of the selected strains

The plant growth-promotion effect of the candidate strains was tested with two different methods: 1) by inoculating onto the root tips of perennial ryegrass seedlings to test direct plant growth-promoting effect of the isolates; 2) via two-compartment petri dishes, where bacteria can only stimulate plant growth through volatile organic compounds (VOCs). When inoculated to root tips, MG27 and MG33 showed more than a 2.5-fold increase on shoot biomass and an approximately two-fold increase on root biomass compared to control. MG43 showed more than a two-fold increase in shoot biomass and no increase in root biomass. Other strains tested did not show any significant plant growth-promotion effect (Additional file 1: Figure S1). When the bacteria were inoculated with two-compartment petri dishes, all tested strains showed significant increases in shoot and root biomass of the perennial ryegrass with variations among strains. MG64 showed an approximately two-fold increase of both shoot and root biomass, while other strains showed more than a 2.5-fold increase of both shoot and root biomass (Additional file 1: Figure S1).

### Genome sequencing of the selected strains and phylogenetic analysis

**Fig. 2:**
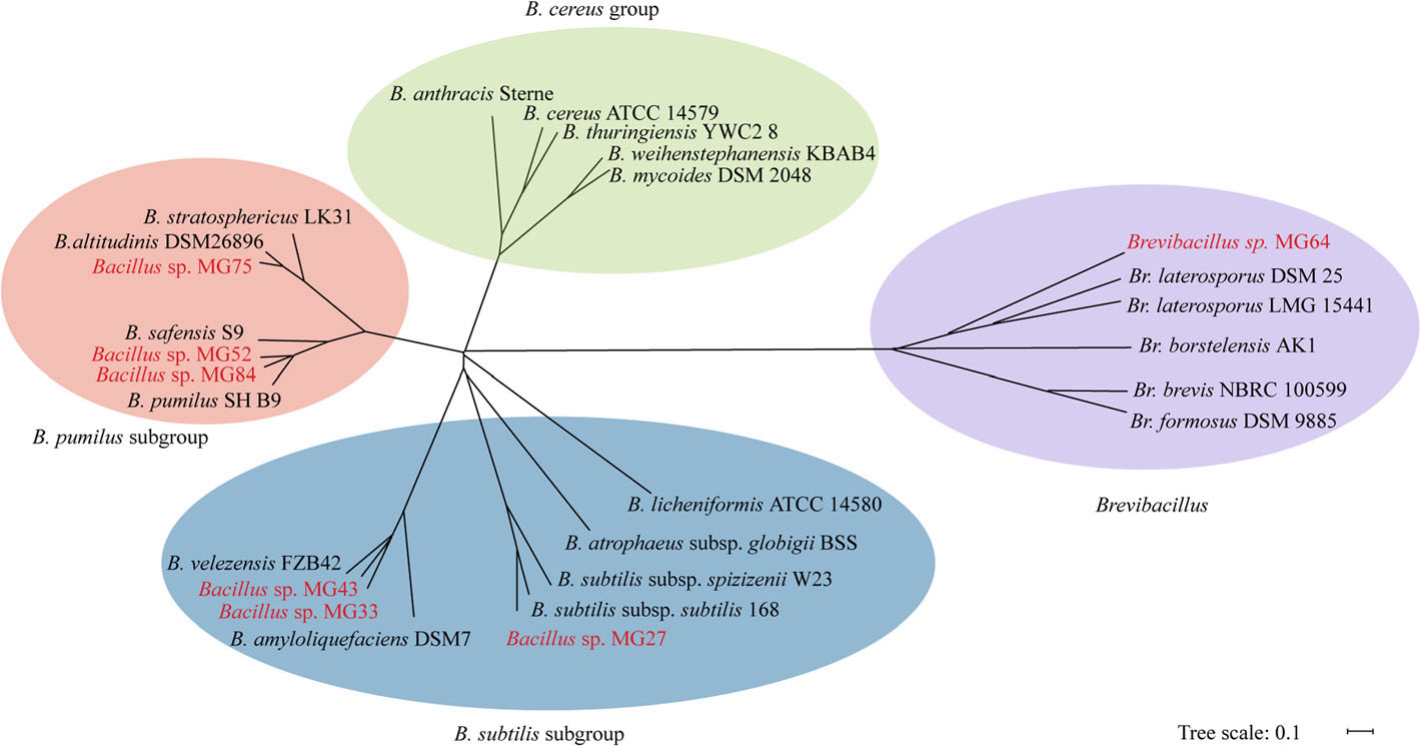
Phylogenetic analysis of the selected strains and their reference strains based on the genomic sequences. The comparison was conducted with Gegenees using a default setting [34]. The dendrogram was constructed in SplitTree [35]. Different groups of bacteria were indicated with different colors of shading. The seven strains isolated in this study were indicated with red font

The genomes of the seven selected strains were sequenced and their DNA sequences were described previously [25]. Phylogenetic analysis using whole-genome sequences was conducted with Gegenees [34] and a phylogenetic tree was built with SplitTree [35]. As presented in Fig. 2, MG27, MG33, and MG43 fall into the *B. subtilis* subgroup, while MG52, MG75, and MG84 belong to the *B. pumilus* subgroup, which is different from 16S rRNA phylogenetic analysis that these two subgroups did not clearly separate (Fig. 1). There are no strains belonging to the group of *B. cereus*, which is in accordance with the 16S rRNA phylogenetic tree (Fig. 1). MG64 was clustered to the genus of *Brevibacillus*, which is far away from the *Bacillus* genus phylogenetically (Fig. 2). The species names of the seven strains were designated as their most closely related strains, namely *B. subtilis* subsp. *subtilis* MG27, *B. velezensis* MG33 and MG43, *B. pumilus* MG52 and MG84, *B. altitudinis* MG75, and *B. laterosporus* MG64.

### Genome mining for BGCs

**Fig. 3:**
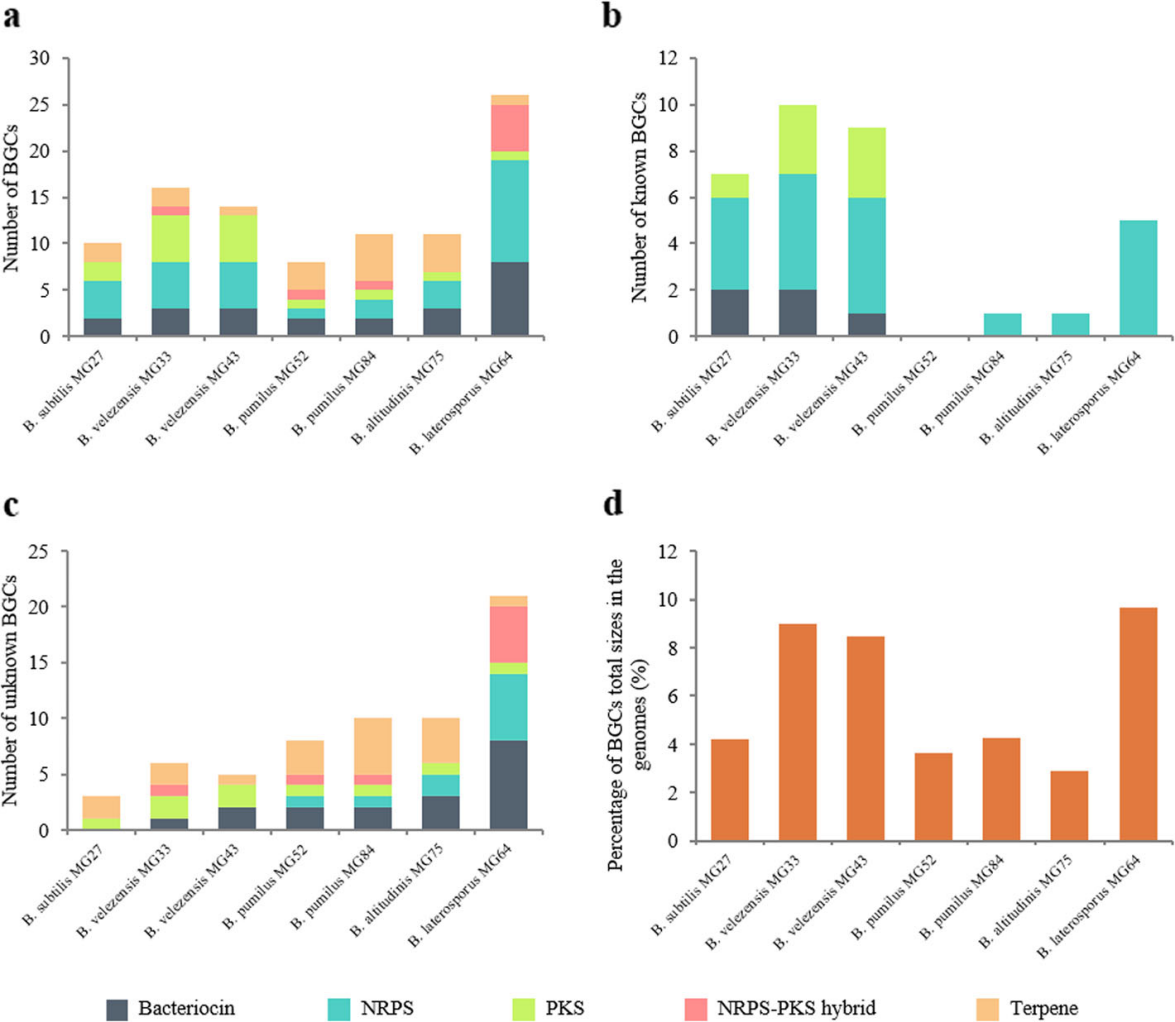
Numbers of BGCs harbored by the strains and the percentage of the total size of BGCs in the genomes. (**a**) total number of BGCs in the strains. (**b**) number of reported BGCs in the genomes of strains. (**c**) number of unknown BGCs found in the strains. BGCs that have different numbers of genes or show less than 70% protein identity to the reported ones were regarded as novel. (**d**) the percentage of BGCs sizes in the genomes

*Bacillus* and closely related species form a great reservoir of antimicrobials [7]. In order to evaluate the biosynthetic potential of the selected strains, their genomic sequences were analyzed by antiSMASH 5.0 [36] for mining of nonribosomal peptide synthetase (NRPS), polyketide synthetase (PKS), NRPS-PKS hybrid, and terpene BGCs, and by BAGEL4 [37] for mining of bacteriocin BGCs. Among the genus of *Bacillus*, the *B. subtilis* subgroup members (*B. subtilis subsp. subtilis* MG27, *B. velezensis* MG33, and *B. velezensis* MG43) harbor abundant NRPSs and PKSs (Fig. 3a) and the majority of the BGCs are assigned to known products (Fig. 3b, Additional file 1: Table S2). The remaining-unknown BGCs from this subgroup are terpene and PKS (Fig. 3c, Additional file 1: Figure S2), including type III PKS, a homodimeric iterative polyketide synthase recently found present in microorganisms [38]. The total size of the BGCs in *B. subtilis subsp. subtilis* MG27 is approximately 176 kb and accounts for 4.2% of the genome size (Fig. 3d). This percentage is in line with the estimation of other *B. subtilis* strains, which is 4–5% on average [39]. *B. velezensis* MG33 and MG43 devote around 8.9 and 8.4% of their genomes to synthesis antimicrobial metabolites, respectively (Fig. 3d). This result is similar to the estimation of *Bacillus velezensis* FZB42, which is 8.5% [40]. The *B. pumilus* subgroup members (*B. pumilus* MG52, *B. pumilus* MG84, and *B. altitudinis* MG75) possess 8 to 11 BGCs (Fig. 3a). The abundance of terpene is an outstanding characteristic of this subgroup (Fig. 3a). Most of the BGCs from this group remain unknown, especially bacteriocins and terpenes (Fig. 3c, Additional file 1: Figure S2, Figure S3). The *B. pumilus* subgroup members devote 2.9 to 4.2% of their genomes to BGCs.

*B. laterosporus* has drawn increasing attention in recent years because of its outstanding ability of antimicrobial production. Borogols [41], brevibacillins [42], tauramamide [43], brevicidine [44], laterocidine [44], etc. are antimicrobials reported in the past two decades. Genome mining reveals that *B. laterosporus* MG64 harbors the most abundant gene clusters among the seven strains, which reach a total number of 26 (Fig. 3a). Five NRPSs were assigned to brevicidine, auriprocine, tyrocidine, petrobactin, bogorol, respectively (Fig. 3b, Additional file 1: Table S2). Up to 21 BGCs in *B. laterosporus* MG64 remained unknown and the majority of them are NRPS, NRPS-PKS hybrid, and bacteriocin (Fig. 3c, Additional file 1: Figure S2, Figure S3). The total size of the BGCs is approximately 500 kb, which accounts for 9.7% of the genome (Fig. 3d). This percentage is higher than *Bacillus velezensis* (8.5%) and *Streptomyces avermitilis* (6.4%), which are well-known antimicrobial producing strains [40, 45]. This result suggests the great value of *B. laterosporus* MG64 in biocontrol and pharmaceutical application, for some of its natural products might have the potential to be antibiotics.

### Potential novel modular BGCs

**Fig. 4:**
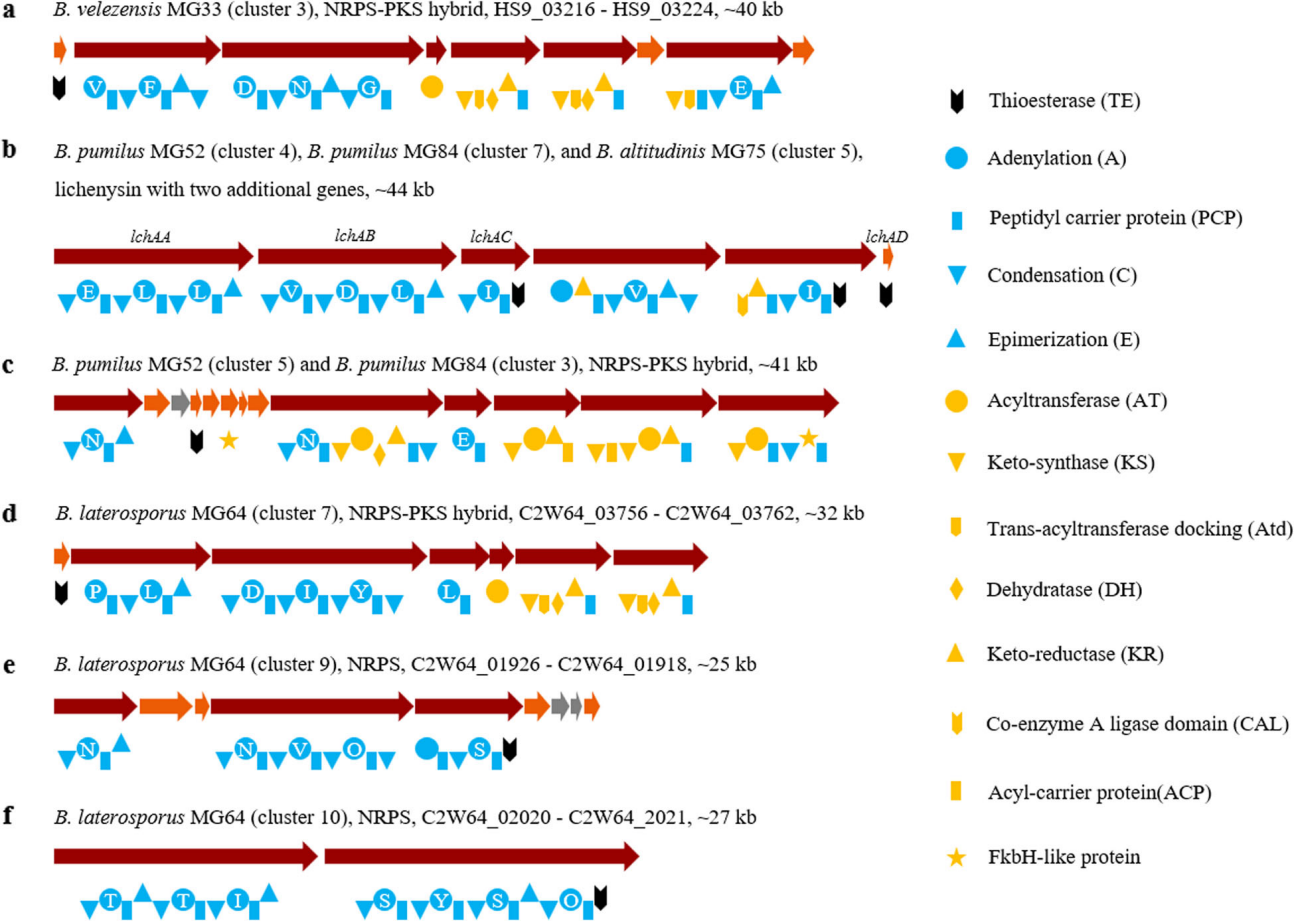
Potential intact and novel BGCs discovered in the genomes of selected strains. (**a**) an NRPS-PKS hybrid discovered in *B. velezensis* MG33. (**b**) a lichenysin-like NRPS-PKS hybrid present in the strains from the *B. pumilus* subgroup. (**c**) an unknown NRPS-PKS hybrid found in both *B. pumilus* MG52 and MG84. (**d**) a potential novel NRPS-PKS hybrid harbored by *B. laterosporus* MG64. (**e**-**f**) two potential novel NRPSs present in *B. laterosporus* MG64. Amino acid residues predicted by antiSMASH was indicated inside the A domains. Cluster number in the brackets corresponding to those in Fig. S2. Genes with different functions are shown in different colors: orange, additional biosynthetic genes; wine-red, core biosynthetic genes; grey, unknown-function genes

NRPS, PKS, and NRPS-PKS hybrid are modular enzymes that synthesize secondary metabolites, some of which are well-known weapons for plant disease control [8]. Modular BGCs found in the selected strains with all essential modules (starting module, elongation module, termination module) were listed in Fig. 4. Despite the abundantly identified BGCs in *B. velezensis* MG33, one modular gene cluster showing no similarity to known BGCs was found (Fig. 4a). This BGC consists of 9 genes and has a total size of 40 kb. The cooccurrence of NRPS domains and PKS domains indicates it is a hybrid of both. The NRPS modules incorporate six amino acid residues while PKS modules likely incorporate and modify one polyketide moiety. It is difficult to predict the potential activity of its final product because the antimicrobials (surfactin, fengycin, bacillomycin D, bacilysin, difficidin, etc.) produced by *B. velezensis* MG33 are well-known for killing different kinds of pathogens [8, 19].

An interesting BGC discovered in *B. pumilus* MG52, *B. pumilus* MG84, and *B. altitudinis* MG75 is partially identical to lichenysin (Fig. 4b), which is a surfactin family lipopeptide biosurfactant produced by *Bacillus licheniformis*. Lichenysin is not only involved in direct pathogen antagonism but also affects the colonization of bacteria to plant, thus regarded as important in biocontrol [8]. This lichenysin-like BGC contains six genes, and four of them showed more than 50% sequence identity to the lichenysin BGC. Furthermore, the amino acid residues incorporated by these four genes are identical to lichenysin as well. However, there are two additional genes in between *lchAC* and *lchAD* (Fig. 4b). They encode four modules, which are responsible for the incorporation of four residues (Fig. 4b). Whether the additional genes are functional or not remains unclear. On the one hand, a thioesterase (TE) domain was encoded by *lchAC*, indicating that the biosynthesis of the lichenysin is likely not affected. On the other hand, other *B. pumilus* strains also showed this interesting phenomenon (data not shown), which suggests this is likely to be an evolutionary horizontal gene transfer. Experimental proofs are needed to answer this interesting question. Another unique NRPS-PKS hybrid BGC in *B. pumilus* MG52 and MG84 contains thirteen genes and encodes ten modules (Fig. 4c). This gene cluster showed 21% similarity to paenilamicin, an antibacterial and antifungal NRPs-PKs hybrid produced by *Paenibacillus larvae* [46]. However, neither the order of genes nor the predicted amino acid composition shows similarity to paenilamicin, indicating the putative novelty of the final product. *B. pumilus* MG52 and MG84 displayed potent activity against bacterial pathogens (Table 1). However, well-known antibacterial compounds were not found by the genome mining (Additional file 1: Table S2). This suggests the potential functionality of these novel BGCs.

Three potential intact and novel BGCs were discovered in *B. laterosporus* MG64 (Fig. 4). The first one is a 32-kb NRPS-PKS hybrid BGC (Fig. 4d). This BGC contains seven genes and encodes six NRPS modules and two PKS modules. This gene cluster does not show any similarity to the reported BGCs, indicating its great novelty. The second one is a 25-kb NRPS that contains nine genes (Fig. 4e). Six modules are encoded by the three core biosynthetic genes, indicates the incorporation of six amino acids. This BGC showed an 11% similarity to zwittermycin A, an NRPs-PKs hybrid produced by *B. cereus* [47]*.* However, the type of BGCs and the gene numbers are different from zwittermycin A, suggesting the putative novelty of the final product. The third one is also identified to be an NRPS (Fig. 4f). This BGC is around 27 kb in size and is constituted with two large core biosynthetic genes, which encode seven modules. The structure of this peptide remained unclear owing to the diverse function of a TE domain [48]. Bogorol and brevicidine that are identified by the genome mining were reported to have antibacterial activity [41, 44]. They are likely responsible for the antibacterial activity of *B. laterosporus* MG64. However, the antimicrobials responsible for its antifungal and antioomycetal activity (Table 1) are not yet clear. Therefore, the three BGCs identified here are potentially functional.

### Potential novel bacteriocin BGCs

**Fig. 5:**
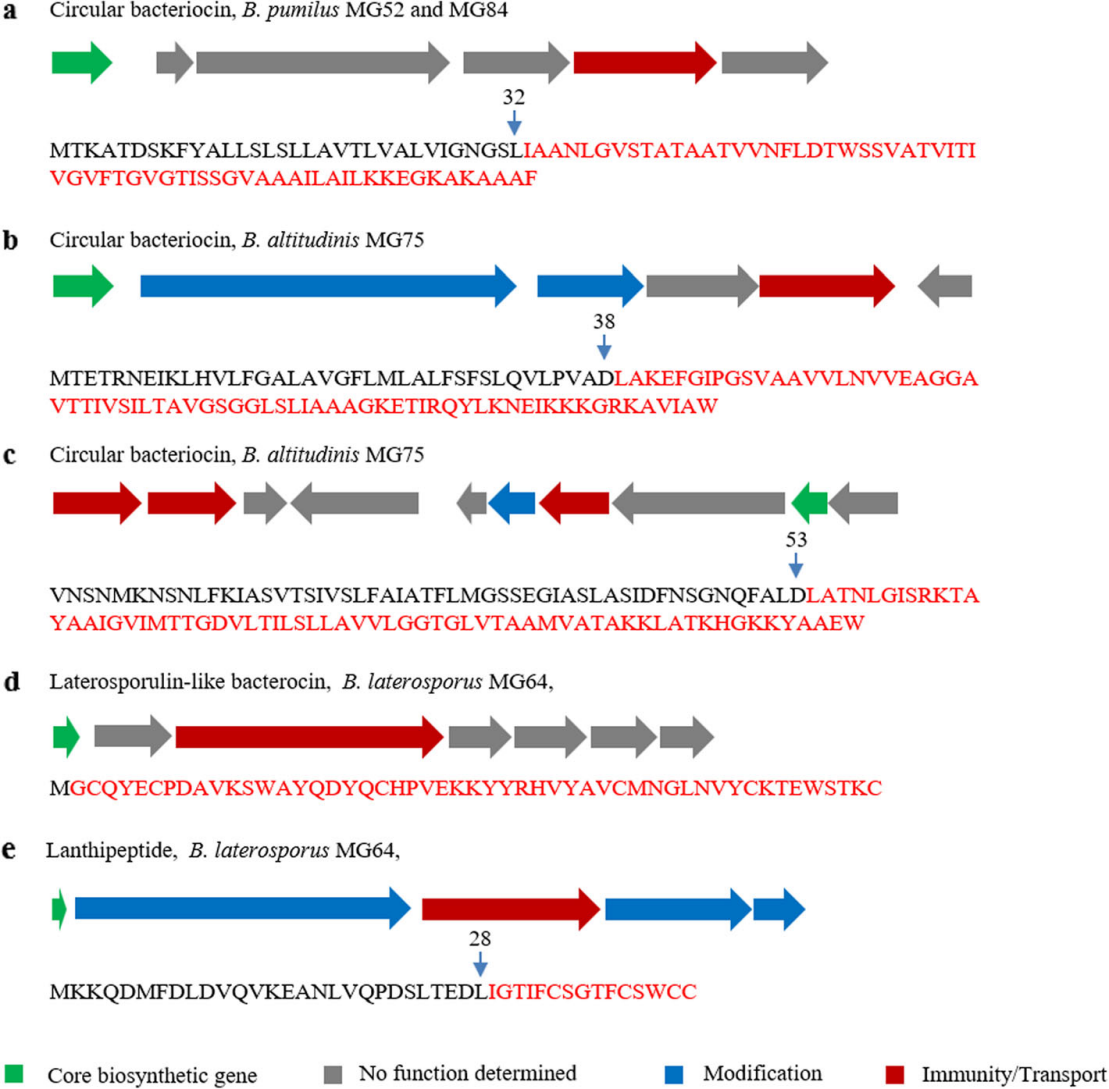
Potential novel bacteriocins with predicted precursor peptides. The BGCs were predicted by BAGEL4 [37]. (**a**) a circular bacteriocin found in both *B. pumilus* MG52 and MG84. (**b**-**c**) two circular bacteriocins discovered in *B. altitudinis* MG75. (**d**) a circular bacteriocin and (**e**) a lanthipeptide harbored by *B. laterosporus* MG64. The potential core peptides are indicated in red. The potential cleavage sites are indicated with arrows. The numbers indicate the position of amino acid residues

Bacteriocins are ribosomally synthesized antimicrobial peptides that mainly kill bacteria closely related to producers. They are classified into three main classes: class I small ribosomally produced and posttranslationally modified peptides (RiPPs), class II unmodified peptides, and class III large antimicrobial peptides (> 10 kDa) [7, 49]. Among them, RiPPs (including lanthipeptides, circular bacteriocins, sactipeptides, linear azole-containing peptides, thiopeptides, glycocins, and lasso peptides) are the most well-studied, wide-distributed, and active peptides [7, 50]. Potential novel RiPPs BGCs with predicted precursors discovered in the selected strains are listed in Fig. 5.

One circular bacteriocin BGC was harbored by both *B. pumilus* MG52 and MG84 (Fig. 5a). The gene cluster contains six genes. The precursor peptide contains 96 amino acids and the C terminus (from I33 to F96) showed 39% identity to amylocyclicin, which is a 6381-Da circular bacteriocin produced by *B. velezensis* FZB42 and showed to be active against closely related Gram-positive bacteria [51]. However, the rest of the genes show very low similarity to amylocyclicin BGC (data not shown), which indicates putative novel modification patterns. The potent activity of *B. pumilus* MG52 and MG84 against the Gram-positive bacterium *S. scabies* (Table 1) and the lack of known antimicrobials in their genomes (Additional file 1: Table S1) suggest the potential activity of this circular bacteriocin.

*B. altitudinis* MG75 harbors two circular bacteriocin BGCs (Fig. 5). The first one constitutes six genes (Fig. 5b). Its precursor peptide contains 108 amino acids and showed 67% protein identity to enterocin AS-48, which is a model circular bacteriocin produced by *Enterococcus* [52]. The second circular bacteriocin from *B. altitudinis* MG75 potentially contains 10 genes (Fig. 5b). The precursor peptide comprises 118 amino acids and the C terminus (from L58 to W118) showed 52% identity to enterocin NKR-5-3B, which is a broad-spectrum antimicrobial produced by *Enterococcus faecium* NKR-5-3 [53]. *B. altitudinis* MG75 did not antagonize the Gram-positive bacterium *S. scabies* (Table1). Therefore, the functionality of these circular bacteriocins remains unclear.

*B. laterosporus* MG64 harbors one circular bacteriocin and one lanthipeptide (Fig. 5). The BGC of circular bacteriocin contains seven genes (Fig. 5d). The core biosynthetic gene encodes a 58-amino acid peptide, which showed 62% protein identity to laterosporulin and 52% to laterosporulin10, both of which are produced by *B. laterosporus* and display antimicrobial activity against several bacterial pathogens [54, 55]. The lanthipeptide BGC from *B. laterosporus* MG64 (Fig. 5e) contains five genes. The second, fourth, and fifth genes were annotated as *lanB* (dehydratase), *lanC* (cyclase), and *lanD* (decarboxylase), respectively, which are commonly found in lanthipeptide BGCs. The core biosynthetic gene encodes a 43-amino acid peptide, which showed 39% similarity to both gallidermin and epidermin, two cationic lanthipeptides produced by *Staphylococcus* and displayed activity against a wide range of Gram-positive bacteria [56]. The antibacterial activity of *B. laterosporus* MG64 is likely conferred by bogorol and brevicidine [41, 44]. Therefore, we cannot elaborate on the putative functions of these bacteriocins.

## Conclusions

In this study, we identified 7 potential PGPR strains, out of 90 strains screened, that can antagonize both phytopathogens and plant-originated mammalian pathogens, thus showing the possibility to employ PGPR to protect the food chain of grass-ruminant-human. Further mining into the genomes of the potential PGPR strains reveals a great number of BGCs, including known and potential novel ones. We show the great potential of *B. pumilus* subgroup strains in bacteriocin and terpene production and the great values of *B. laterosporus* MG64 in the production of natural products, which may also have pharmaceutical potential. Furthermore, eleven potential intact and novel BGCs were analyzed in detail, including two NRPSs, four NRPS-PKS hybrids, and five bacteriocins (four circular bacteriocins and one lanthipeptide). Further efforts will be directed to identify these interesting secondary metabolites as well as their contribution to biocontrol.

## Methods

### Plant material, bacterial isolation, and strains

Perennial ryegrass seeds (cultivar Barsprinter) used in this study were provided by the company Barenbrug in Nijmegen, the Netherlands. Bacterial isolation from rhizosphere soil samples has been described previously and the genomic sequences of the most promising strains (MG27, MG33, MG43, MG52, MG64, MG75, and MG84) were placed in GenBank under accession no. of QJJA00000000, QJJB00000000, QJJC00000000, QJIZ00000000, QJJD00000000, QIMF00000000, and QJJE00000000, respectively [25]. The seven promising strains were also deposited in the NCCB collection (the Netherlands) under the accession numbers from NCCB100736 to NCCB100742.

### Identification of bacterial strains

The cells of each bacterial isolate were collected by centrifuging at 10,000 rpm for 1 min and suspending in Mili-Q water. The suspension was heated at 100 °C for 10 min and centrifuged at 10,000 rpm for 1 min after cooling down to room temperature. The supernatant was used as template DNA in a PCR to amplify 16S rRNA for characterization. PCR amplifications were conducted with bacterial-specific 16S rRNA primers 27F (5′-AGAGTTTGATCMTGGCTCAG-3′) and 1492R (5′-CGGTTACCTTGTTACGACTT-3′) as well as the high fidelity Phusion polymerase (Thermo Fisher Scientific). PCR products were purified with a NucleoSpin Gel and PCR Clean-up kit (Macherey-Nagel) and sequenced at Macrogen Inc. The resulted partial sequences of 16S rRNA were aligned with relevant type strains with Muscle [57] in MEGA7 [26]. A neighbor-joining consensus tree [58] was constructed based on the alignment. The main parameters used were as follows: Bootstrap method and 1000 bootstrap replications for phylogeny test [59], Tamura-Nei model for nucleotide substitution [60], Gamma distribution for rate variation among sites, and complete deletion for treatment of gaps. The resulted phylogenetic tree was visualized and modified in iTOL [61].

### In vitro antagonistic assay

Bacterial pathogens were streaked on LB plates and incubated at 28 °C overnight. The colonies were suspended in LB broth and mixed with melted LB medium (cool down to 45 °C) at a final concentration of 1 × 10^− 6^ CFU/ml before pouring plates. After solidification, 5 μl bacterial solution (OD_600_ = 1.0) made with isolates was inoculated onto the plate. The plates were incubated at 28 °C for 48 h before measuring the diameters of inhibition halos. Fungal and oomycetal pathogens were inoculated on TSA plates (TSB solidified with 1.5% agar) and incubated at 28 °C for 5 days. An agar plug (5 mm diameter) with fungal hyphae or oomycete spore was cut and inoculated onto the center of a new TSA plate. A 5 μL sample of each bacterial solution at an OD_600_ of 1.0 was spotted 2 cm away from the plug symmetrically. Plates were double sealed with parafilm and incubated at 28 °C for another 5 days. The antagonistic activities were then documented.

### Plant growth-promoting assay

For surface sterilization, the ryegrass seeds were pre-treated with 0.3 M HCl for 6 h, followed by submerging in 2% sodium hypochlorite for 5 min and then washed with sterile water for 10 times to remove HCl and sodium hypochlorite completely. The seeds were germinated on wet sterile filter paper in a large petri-dish which was sealed with parafilm and then incubated at 25 °C without light. After germination for 5 days, the ryegrass seedlings were then transferred to fresh ½ MS (Duchefa Biochemie) plates solidified with 0.8% plant agar (Duchefa Biochemie). After 2 days growing in ½ MS plates, 5 μl bacterial solution in 10 mM MgSO_4_ with an OD_600_ of 1.0 was inoculated, while the same amount of 10 mM MgSO_4_ solution was used as control. For root tip inoculation assays, the bacterial solution was inoculated to the root tip of each seedling. To study the volatile effect, the bacterial solution was inoculated onto the center of a small LB agar plate that forms a physically separated compartment inside the ½ MS medium plate. After co-culture for 7 days. The ryegrass seedlings were harvested and the biomass of shoot and root were measured separately. A one-way ANOVA analysis using a Tukey post-hoc test was conducted with SPSS (*P* < 0.05) to evaluate the significance.

### Genome sequencing and phylogenetic analysis

The genome sequences of the selected strains were determined as described previously [25]. Genome-scale comparison of the seven bacterial strains and other relevant strains were conducted with Gegenees [34] based on a fragmented nucleotides alignment with a setting of 200/100. Based on the alignment, a dendrogram was constructed in SplitTree 4.14.4 [35] with a neighbor-joining method and visualized in iTOL [61].

### Genome mining for BGCs

The genome mining for biosynthetic gene clusters of antimicrobial compounds including NRPs, PKs, NRPs-PKs hybrids, bacteriocins, and terpenes was conducted with antiSMASH 5.0 [36] and BAGEL4 [37]. Each draft genome was assembled into a pseudomolecule using a closely related strain as a reference before applying to the pipelines. The genes predicted from both pipelines were further confirmed with protein BLAST. BGCs that have different numbers of genes or show less than 70% protein identity to the reported ones were regarded as novel.

## Supplementary information

**Additional file 1 : Table S1.** Screening of bacterial isolates for antagonistic strains. **Table S2.** Known antimicrobial BGCs found in the genomes of isolates. **Figure S1.** Plant growth-promotion effects of the selected strains on perennial ryegrass. **Figure S2.** Potential novel NRPS, PKS, NRPS-PKS hybrid, terpene BGCs mined from AntiSMASH 5.0. **Figure S3.** Potential novel bacteriocin BGCs mined from BAGEL4.

## Data Availability

The genomic sequences of the seven selected strains are publicly available in DDBJ/EMBL/GenBank. Their accession numbers are QJJA00000000, QJJB00000000, QJJC00000000, QJIZ00000000, QJJD00000000, QIMF00000000, and QJJE00000000. The seven selected strains were deposited in the NCCB collection (the Netherlands) under the accession numbers from NCCB100736 to NCCB100742.

## Abbreviations

A: Adenylation
ACP: Acyl-carrier protein
AT: Acyltransferase
Atd: Trans-acyltransferase docking
BGCs: Biosynthetic gene clusters
C: Condensation
CAL: Co-enzyme A ligase domain
DH: Dehydratase
E: Epimerization
ISR: Induced systemic resistance
KR: Keto-reductase
KS: Keto-synthase
NRPs: Nonribosomal peptides
NRPSs: Nonribosomal peptide synthetases
PCP: Peptidyl carrier protein
PGPR: Plant growth-promoting rhizobacteria
PKs: Polyketides
PKSs: Polyketide synthetases
RiPPs: Ribosomally produced and posttranslationally modified peptides
TE: Thioesterase
VOCs: Volatile organic compounds
