# Supplementary material for: Characterization of plant growth-promoting rhizobacteria from perennial ryegrass and genome mining of novel antimicrobial gene clusters
Source: BMC Genomics. 2020 Feb 12;21:157. doi: 10.1186/s12864-020-6563-7 (PMC7017464; doi:10.1186/s12864-020-6563-7)
Supplement: Supplementary file 1 — Additional file 1 : Table S1. Screening of bacterial isolates for antagonistic strains. Table S2. Known antimicrobial BGCs found in the genomes of isolates. Figure S1. Plant growth-promotion effects of the selected strains on perennial ryegrass. Figure S2. Potential novel NRPS, PKS, NRPS-PKS hybrid, terpene BGCs mined from AntiSMASH 5.0. Figure S3. Potential novel bacteriocin BGCs mined from BAGEL4. [file 12864_2020_6563_MOESM1_ESM.docx]

Characterization of plant growth-promoting rhizobacteria from perennial ryegrass and genome mining of novel antimicrobial gene clusters

Zhibo Li^1^, Chunxu Song^1a^, Yanglei Yi^1b^, Oscar P. Kuipers^1^*

^1^ Department of Molecular Genetics, University of Groningen, Groningen, the Netherlands

^a^ College of Resources and Environmental Sciences; National Academy of Agriculture Green Development; Key Laboratory of Plant-Soil Interactions, Ministry of Education, China Agricultural University, 100193 Beijing, China

^b^ Present address: College of Food Science and Engineering, Northwest A&F University, Yangling, Shaanxi, China.

* Author of correspondence:

Oscar P. Kuipers, tel:+31 50 3632093; email: [o.p.kuipers@rug.nl](mailto:o.p.kuipers@rug.nl)

**Table S1.** Screening of bacterial isolates for antagonistic strains

| **Genus** | **Group** | **Strain** | **Activity** | | **Strain** | **Activity** | | **Strain** | **Activity** | |
| --- | --- | --- | --- | --- | --- | --- | --- | --- | --- | --- |
|  |  |  | **Xtg** | **Mo** |  | **Xtg** | **Mo** |  | **Xtg** | **Mo** |
| *Bacillus* | *B. subtilis* group | MG18 | - | + | MG55 | + | + | MG69 | - | - |
|  |  | MG27 | + | + | MG56 | + | + | MG73 | + | - |
|  |  | MG28 | + | + | MG57 | + | + | MG74 | + | + |
|  |  | MG32 | + | + | MG58 | + | - | MG75 | + | - |
|  |  | MG33 | + | + | MG59 | + | - | MG77 | + | - |
|  |  | MG37 | + | + | MG60 | - | - | MG79 | + | - |
|  |  | MG39 | + | + | MG61 | - | + | MG82 | + | + |
|  |  | MG42 | + | + | MG62 | - | + | MG84 | + | - |
|  |  | MG43 | + | + | MG63 | - | + | MG85 | - | + |
|  |  | MG44 | - | - | MG65 | + | + | MG89 | - | - |
|  |  | MG51 | + | + | MG66 | - | - | MG90 | - | + |
|  |  | MG52 | + | - | MG67 | + | + |  |  |  |
|  |  | MG53 | + | - | MG68 | - | - |  |  |  |
|  | *B. cereus* group | MG4 | - | - | MG22 | - | - | MG70 | - | - |
|  |  | MG10 | - | - | MG23 | - | - | MG71 | - | - |
|  |  | MG11 | - | + | MG25 | + | - | MG72 | + | - |
|  |  | MG14 | - | - | MG26 | - | - | MG78 | - | - |
|  |  | MG15 | - | - | MG30 | - | - | MG80 | - | - |
|  |  | MG16 | - | - | MG31 | - | - | MG81 | + | - |
|  |  | MG17 | - | - | MG34 | - | - | MG83 | - | - |
|  |  | MG19 | - | - | MG38 | - | - | MG86 | - | - |
|  |  | MG20 | - | - | MG46 | - | - | MG87 | - | - |
|  |  | MG21 | - | - | MG48 | - | - | MG88 | - | - |
|  | *B. megaterium* group | MG1 | - | - | MG9 | - | - | MG41 | - | - |
|  |  | MG2 | - | - | MG13 | - | - | MG45 | - | - |
|  |  | MG3 | - | - | MG29 | - | - | MG47 | - | - |
|  |  | MG6 | - | - | MG35 | - | - | MG49 | + | - |
|  |  | MG7 | - | - | MG36 | - | - |  |  |  |
|  |  | MG8 | - | - | MG40 | - | - |  |  |  |
| *Lysinibacillus* | - | MG5 | - | - | MG24 | - | - |  |  |  |
|  |  | MG12 | - | - | MG50 | - | - |  |  |  |
| *Solibacillus* | - | MG54 | - | - | MG76 | - | - |  |  |  |
| *Brevibacillus* | - | MG64 | + | + |  |  |  |  |  |  |

Xtg is short for *X. translucens* pv*. graminis*. Mo stands for *M. oryzae*. The selected strains are indicated in red. ‘+’ means clear inhibition halo was observed while ‘-’ indicates no activity.

**Table S2.** Known antimicrobial BGCs found in the genomes of selected strains

| Strain | Locus tag in NCBI | Size (bp) | Type | Antimicrobial compound |
| --- | --- | --- | --- | --- |
| *B. subtilis* MG27 | HS3_01760 - HS3_01767 | 7284 | NRPS | Bacilysin |
|  | HS3_01800 - HS3_01807 | 6939 | Bacteriocin | Subtilosin A |
|  | HS3_02375 - HS3_02387 | 19594 | NRPS | Bacillibactin |
|  | HS3_02542 - HS3_02557 | 77097 | PKS | Bacillaene |
|  | HS3_03507 - HS3_03511 | 8075 | Bacteriocin | Subtilomycin |
|  | HS3_03670 - HS3_03673 | 26146 | NRPS | Surfactin |
|  | HS3_04320 - HS3_04321 | 7516 | NRPS | Fengycin (partial) |
|  | HS3_04464 - HS3_04466 | 7668 | NRPS | Fengycin (partial) |
| *B. velezensis* MG33 | HS9_00027 - HS9_00030 | 37245 | NRPS | Bacillomycin D (Iturin) |
|  | HS9_00297 - HS9_00308 | 70109 | PKS | Bacillaene |
|  | HS9_00628 - HS9_00636 | 53268 | PKS | Macrolactin |
|  | HS9_02290 - HS9_02296 | 7298 | NRPS | Bacilysin |
|  | HS9_03348 - HS9_03351 | 26159 | NRPS | Surfactin |
|  | HS9_03453 - HS9_03455 | 9288 | Bacteriocin | Amyloliquecidin |
|  | HS9_03812 - HS9_03824 | 19408 | NRPS | Bacillibactin |
|  | HS9_03966 - HS9_03972^*^ | 72103 | PKS | Difficidin |
|  | HS9_02738 - HS9_02740^*^ |  |  |  |
|  | HS9_03835 - HS9_03839 | 3934 | Bacteriocin | Amylocyclicin |
|  | HS9_02779 - HS9_02780 | 11419 | NRPS | Fengycin (partial) |
|  | HS9_02781 | 3672 | NRPS | Fengycin (partial) |
|  | HS9_00002 - HS9_00003 | 7536 | NRPS | Fengycin (partial) |
| *B. velezensis* MG43 | C2W63_00620 - C2W63_00629 | 54848 | PKS | Macrolactin |
|  | C2W63_00883 - C2W63_00894 | 70106 | PKS | Bacillaene |
|  | C2W63_01016 - C2W63_01019 | 37286 | NRPS | Bacillomycin D (Iturin) |
|  | C2W63_01638 - C2W63_01649 | 66186 | PKS | Difficidin |
|  | C2W63_02562 - C2W63_02568 | 7298 | NRPS | Bacilysin |
|  | C2W63_03182 - C2W63_03187 | 4173 | Bacteriocin | Amylocyclicin |
|  | C2W63_03197 - C2W63_03209 | 19410 | NRPS | Bacillibactin |
|  | C2W63_03554 - C2W63_03556^*^ | 26159 | NRPS | Surfactin |
|  | C2W63_01267 - C2W63_01268 | 11908 | NRPS | Fengycin (partial) |
|  | C2W63_01269 | 3789 | NRPS | Fengycin (partial) |
|  | C2W63_01043 - C2W63_01044 | 8025 | NRPS | Fengycin (partial) |
| *B. altitudinis* MG75 | US8_00456 - US8_00461 | 6103 | NRPS | Bacilysin |
| *B. pumilus* MG84 | C2W59_01112 - C2W59_01118 | 12865 | NRPS | Bacillibactin |
| *B. laterosporus* MG64 | C2W64_00357 - C2W64_00362 | 55318 | NRPS | Brevicidine |
|  | C2W64_03725 - C2W64_03728 | 41606 | NRPS | Auriprocine |
|  | C2W64_03774 - C2W64_03778 | 37314 | NRPS | Tyrocidine |
|  | C2W64_04196 - C2W64_04201 | 7104 | NRPS | Petrobactin |
|  | C2W64_00401 - C2W64_00410 | 54323 | NRPS | Bogorol |

Known BGCs were not found for the strain of *B. pumilus* MG52.

* only these genes can be found in the NCBI database, but the whole operon was found in the pseudomolecule that is assembly with all contigs using close related strains as a template.


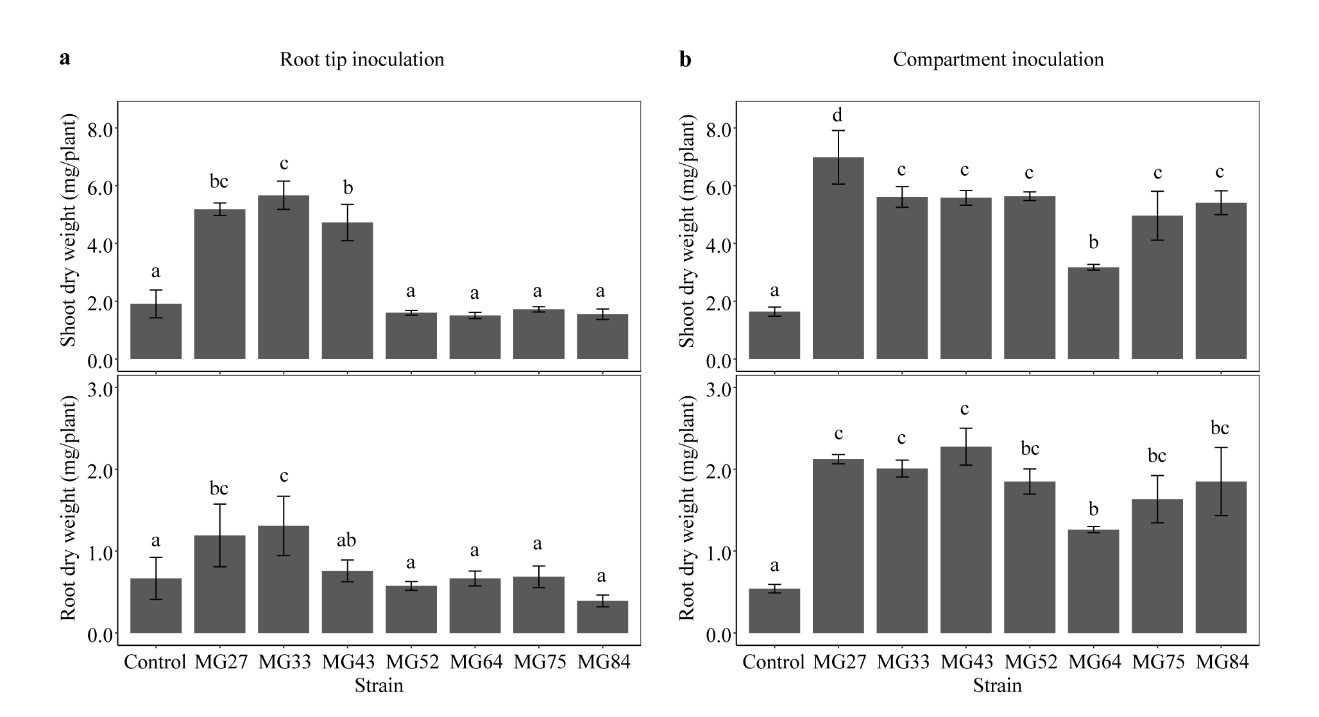


**Figure S1.** Plant growth-promotion effects of the selected strains on perennial ryegrass. (**a**) plant growth-promotion effect of root-tip inoculation of bacteria. (**b**) effect of VOCs produced by candidate strains on the growth of perennial ryegrass. Sterile water was used as a control. Three replicates were used for each treatment. Different letters indicate significant differences between treatments (One-way ANOVA, Tukey post-hoc test, *P* < 0.05).


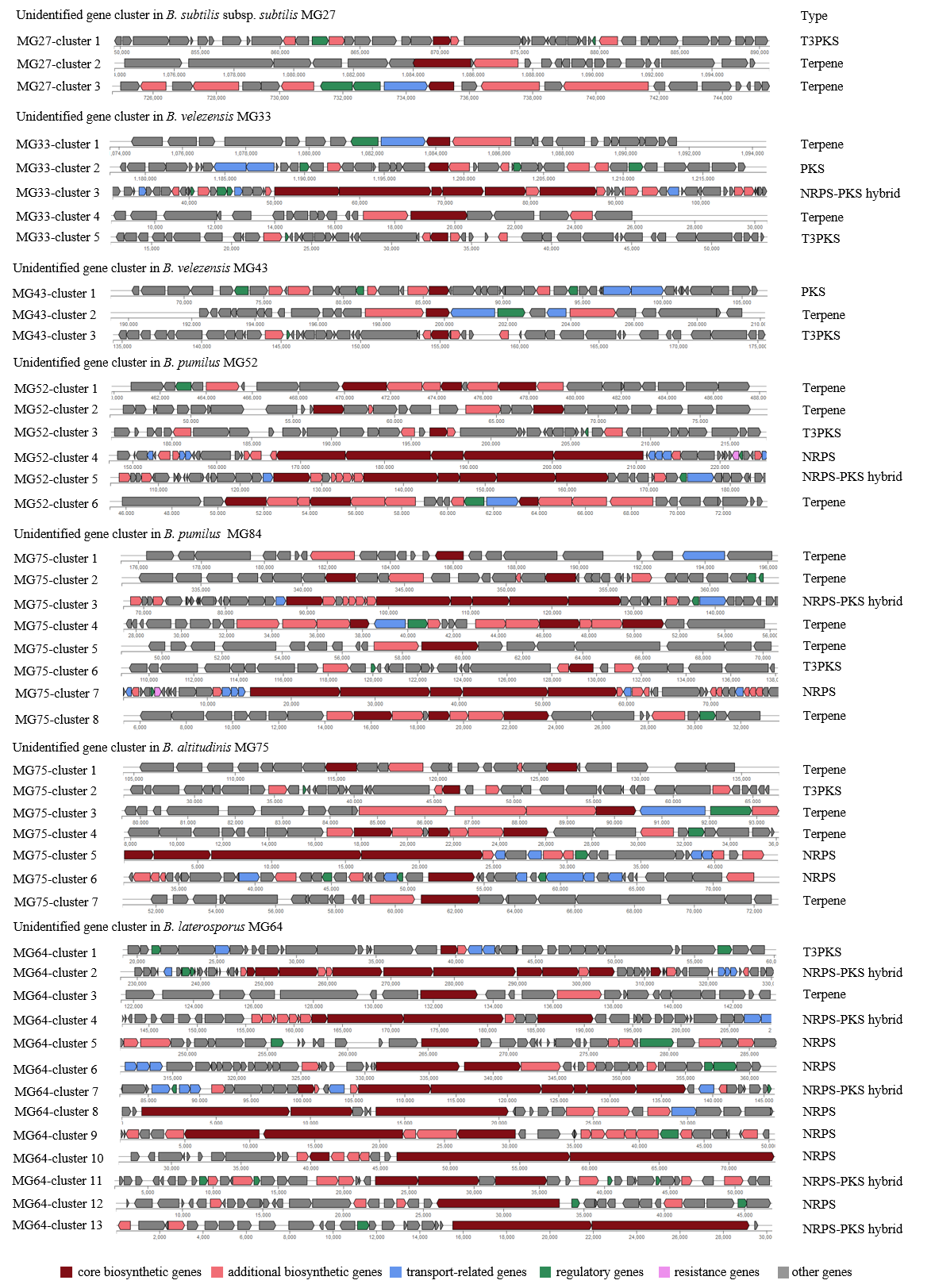


**Figure S2.** Potential novel NRPS, PKS, NRPS-PKS hybrid, terpene BGCs mined from AntiSMASH 5.0. Each draft genome was assembled into a pseudomolecule using a closely related strain as a reference before applying to the pipeline. BGCs that have different numbers of genes or show less than 70% protein identity to the reported ones were regarded as novel. Multiple gene clusters in the same call from antiSMASH were carefully analyzed.


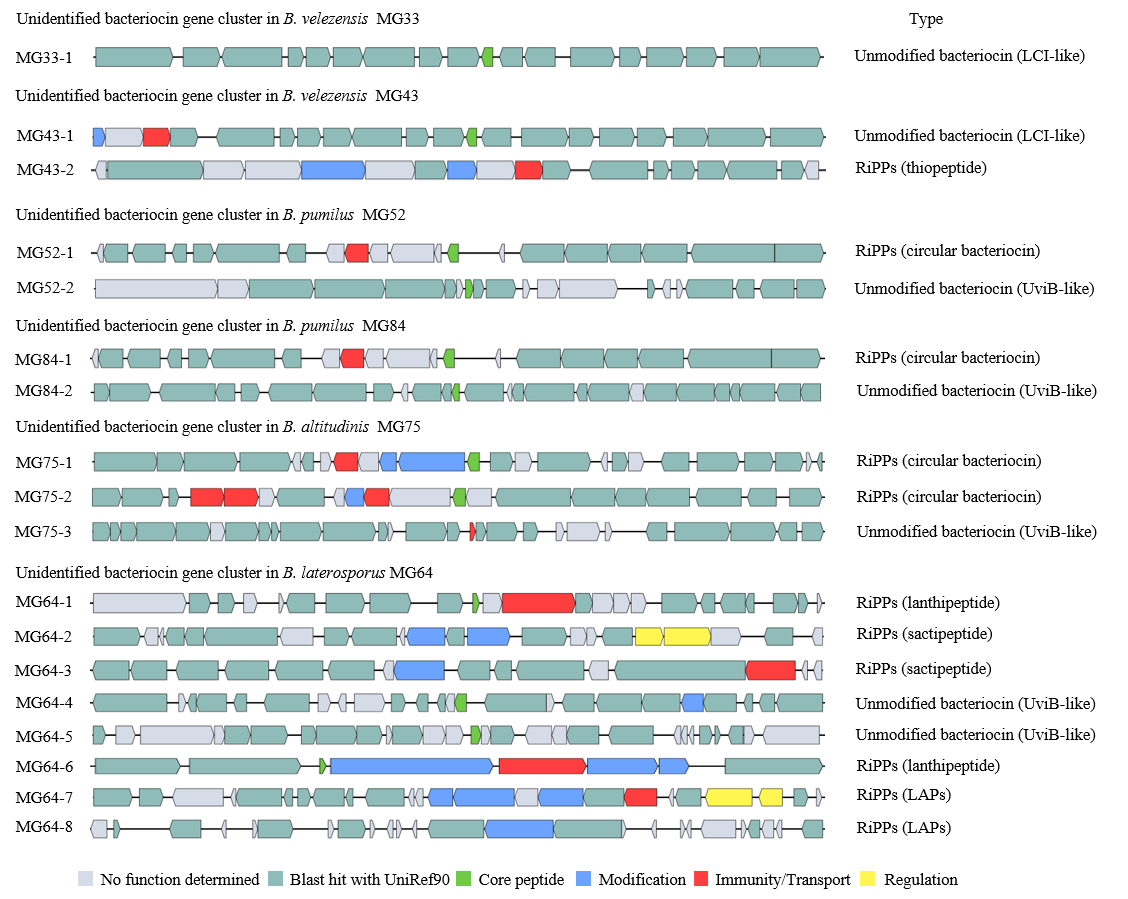


**Figure S3.** Potential novel bacteriocin BGCs mined from BAGEL4. Each draft genome was assembled into a pseudomolecule using a closely related strain as a reference before applying to the pipeline. BGCs that have different numbers of genes or show less than 70% protein identity to the reported ones were regarded as novel.
